# Comfrey and Buttercup Eaters: Wild Vegetables of the Imereti Region in Western Georgia, Caucasus

**DOI:** 10.1007/s12231-017-9379-x

**Published:** 2017-05-18

**Authors:** Łukasz Łuczaj, Boris Tvalodze, David Zalkaliani

**Affiliations:** 1 Department of Botany, Institute of Biotechnology, University of Rzeszów, Werynia 502, 36-100 Kolbuszowa, Poland; 2 Kutaisi Botanic Garden, #2 Leselidze St., 4600 Kutaisi, Georgia

## Introduction

The use of wild greens is an important issue in gastronomic ethnobotany as in some parts of the world, wild greens have been widely used to supplement human nutrition (Bharucha and Pretty 2010; Cruz-Garcia and Struik 2015; Johns 1990; Leonti 2012; Serrasolses et al. 2016; Turner et al. 2011). One of the places where the use of wild vegetables has been sparsely documented until recently, in spite of the incredible richness of their use, is the area of the Caucasus. Some uses of wild vegetables in this area are recorded by older Russian and Georgian sources (see for example Grossgejm 1952; Javakhishvili 1986) and a few general ethnobotanical studies were made recently (Bussmann et al. 2014, 2016a, b, 2017a, b; Hovsepyan et al. 2016), whereas Kaliszewska and Kołodziejska-Degórska (2015) studied the use of wild vegetables in Dagestan (North Caucasus, Russian Federation). However, no such studies have been conducted in the Imereti region. We document the use of all the wild greens, which are predominantly used in a dish called —which according one of the Georgian transliteration rules (Romanization system for Georgian link 2017) is written pkhali (though in some texts it is also written pxali, phkhali, phali, or pchali). This dish is of great importance in the culinary tradition of Georgia, especially its western part, and is eaten almost on a daily basis. Pkhali is also made with cultivated vegetables such as cabbage (*Brassica oleracea* L.), beetroot (*Beta vulgaris* L.), or spinach (*Spinacia oleracea* L.), but the consumption of pkhali made of wild vegetables, so called *veluri pkhali* (wild pkhali) or *mindvris pkhali* (field pkhali), is equally common. Pkhali constitutes the main form of consumption of wild greens in the area and is served as a side dish. The term *mkhali*, the literary version, is often used as well, while pkhali is its synonym in the local dialects of Imereti, Guria, and Racha (Lomtatidze et al. 1962). As a large number of species are used in the dish, some of them of little known edibility, it is of scientific and economic importance to document the plants used.

## Study Site

Imereti is a historical region of western Georgia located on the Colchic Plain, sandwiched between the Great and Lesser Caucasus Mountains. The climate is transitional between humid subtropical and warm temperate, with high rainfall throughout the year. Imereti is a plain with some low mountains surrounding it. The climate is transitional between humid subtropical and warm temperate, with high rainfall throughout the year (Kordzakhia 1971). The mean August temperature is 29 °C (the hottest month), and in January, it is 8 °C. In this climate, some wild vegetables may be collected virtually all year, as a clear drought period is not apparent. The native vegetation is composed of deciduous forests, and the dominant trees are *Quercus robur* spp. *imeretina* (Steven ex Woronow) Menitsky, *Zelkova carpinifolia* (Pall.) K. Koch, *Carpinus betulus* L., *Castanea sativa* L., *Alnus glutinosa* ssp. *barbata* (C.A.Mey.) Yalt, *Corylus avellana* L., *Acer cappadocicum* Gled., *Fagus orientalis* Lipsky, *Ulmus glabra* Huds., *Buxus colchica* Pojark., and *Prunus laurocerasus* L. Large tracts of the Caucasian foothills are managed as wood pastures with freely roaming cattle and pigs, and many species of fruit trees are interspersed between deciduous copses and pastures (Nakhutsrishvili 2012; Otte et al. 2011; Zazanashvili et al. 2000). The area is relatively densely populated. The local farmers plant a variety of annual crops, mainly maize, and there are multi-species orchards around each house.

Kutaisi is the largest town in Imereti (196,000 inhabitants) and the third most populated town in Georgia. It has two large vegetable markets. The one in the center of the city is a retail market including wild vegetables, while the wholesale market is located outside the city center and hosts less wild vegetables. Single wild vegetable stalls may also be encountered in smaller towns, at least occasionally, particularly in Samtredia and Choni.

## Methods

We carried out 41 single and group interviews among knowledgeable informants (40 women, 13 men), selected mainly through contacts with village leaders and by the snowball technique between March and June 2016. The informants were usually accompanied by their extended families who commented on the information and supplied specimens. The informants supplied data about wild vegetable use in the following towns and villages: Bagdati, Banoja, Cholebi, Geguti, Gelati, Gumbra, Khoni, Kumistavi, Kutaisi, Maglaki, Meskheti, Mukhiani, Opshkviti, Partskhanakanevi, Rioni, Sakhulia, Samtredia, Simoneti, Sormoni, Tkibuli/Hresili, Vani, Vartsikhe, Zarati, and Zubi. The age of respondents ranged from 42 to 85 (mean 65, median 66 years). In the interviews, we asked which wild plants were added to the pkhali dish. We also asked interviewees to list other leaves, fruits, roots, or mushrooms used for food or herbal drinks in order to see wild vegetables in the context of all wild food. However, for this paper, we only list the numbers of species listed in other food categories without specifying the species. Voucher specimens were deposited in the herbarium of the Faculty of Biology, University of Warsaw in Poland (WA). The International Society of Ethnobiology Code of Ethics (2006) was followed (see website link).

## Results

On average, respondents mentioned 10.4 species of wild greens per interview (compared to 6.9 species of fruits and 6.3 species of fungi). Altogether, 53 species of wild green vegetables were documented (Table 1). Vegetables for pkhali are boiled for 10 to 30 min, then strained and minced or finely chopped. They are added to crushed or minced walnuts and spiced with vinegar, dill (*Anethum graveolens* L.), coriander (*Coriandrum sativum* L.), pennyroyal (*Mentha pulegium* L.), celery (*Apium graveolens* L.), and parsley (*Petroselinum crispsum* (Mill.) (Fuss). Some more abundant wild vegetables are made as single-species dishes, but most species are used in a mix, and there is no general rule as to which species are served single and which separately. A form of pkhali is also made without walnuts, in which the green mass is spiced by *tkemali*, a sauce made of green cherry plums (*Prunus cerasifera* Ehrh. s.l.) and spiced with similar herbs as the classic walnut pkhali. Mixed plants for pkhali are commonly sold in Kutaisi in the main city market, where 5 to 15 sellers may be encountered every day from the beginning of the year until late April, with a few still selling the plants until June.

**Table 1:**
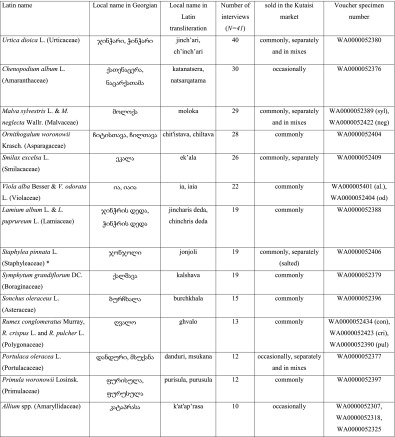

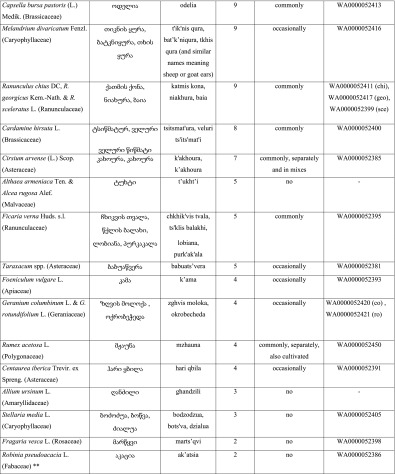

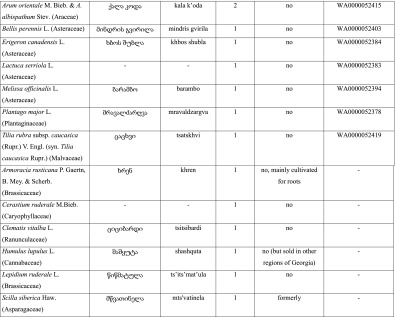
Species of wild plants used in the dish known as pkhali

## Discussion

We recorded five species which are not listed in inventories of wild edible plants (e.g., Hedrick 1919; Tanaka 1976; Kunkel 1984; Plants for a Future 2017), nor are they listed in any ethnobotanical literature concerning wild foods. These are *Ranunculus chius* DC, *Ranunculus georgicus* Kem.-Nath., *Symphytum grandiflorum* DC, *Geranium columbinum* L., and *Geranium rotundifolium* L. It must be emphasized that the way wild vegetables are consumed in Georgia, i.e., with crushed walnuts, is very unique to this country. It is interesting that many toxic wild vegetables, such as buttercups *Ranunculus* spp. and comfrey *S*. *grandiflorum*, are used and sold in the market of Kutaisi. Raw buttercups contain protoanemonin, (Aslam and Ijaz 2012) which is very pungent, and *Symphytum* species contain pyrrholizidine (PA) alkaloids (e.g., Rode 2002; Roitman 1981). Prolonged cooking probably removes most of these toxins, but there is a lack of studies focused specifically on the alimentary use of comfrey after longer cooking.
